# Recent advances in our understanding of the primate corticospinal system

**DOI:** 10.12688/f1000research.17445.1

**Published:** 2019-03-11

**Authors:** Roger Lemon

**Affiliations:** 1 Department of Clinical and Motor Neuroscience, Queen Square Institute of Neurology, Box 28 National Hospital, Queen Square, London, WC1N 3BG, UK

**Keywords:** primate, corticospinal, cortex, motor, movement, dexterity, tool-use, ALS

## Abstract

The last few years have seen major advances in our understanding of the organisation and function of the corticospinal tract (CST). These have included studies highlighting important species-specific variations in the different functions mediated by the CST. In the primate, the most characteristic feature is direct cortico-motoneuronal (CM) control of muscles, particularly of hand and finger muscles. This system, which is unique to dexterous primates, is probably at its most advanced level in humans. We now know much more about the origin of the CM system within the cortical motor network, and its connectivity within the spinal cord has been quantified. We have learnt much more about how the CM system works in parallel with other spinal circuits receiving input from the CST and how the CST functions alongside other brainstem motor pathways. New work in the mouse has provided fascinating insights into the contribution of the CM system to dexterity. Finally, accumulating evidence for the involvement of CM projections in motor neuron disease has highlighted the importance of advances in basic neuroscience for our understanding and possible treatment of a devastating neurological disease.

The cortico-motoneuronal (CM) system is unique to dexterous primates. It provides a direct pathway from motor cortex to the alpha motoneuron. It has long been associated with skilled use of the hands and with tool-making in particular ^1^. Recent advances have allowed a better definition of its origin within the cortical network and its connectivity within the spinal cord.

## ‘New’ M1: the origin of the cortico-motoneuronal output

We know from the work of Rathelot and Strick ^2, 3^, using retrograde transneuronal tracers, that in the macaque monkey, CM neurons are found in two main cortical areas: ‘new’ M1 and area 3a, part of primary somatosensory cortex (S1). Primary motor cortex (M1) is the same as Brodmann area 4; Rathelot and Strick coined the term ‘new’ M1 to define the caudal area of M1 which is restricted to the anterior bank of the central sulcus; very few CM neurons were found more rostrally in the ‘old’ M1 region, on the convexity of the gyrus. This rostral region also gives rise to corticospinal projections, projections that do not terminate on motoneurons, and it also projects to the pontine nuclei and brainstem centres, giving rise to descending motor pathways, including the reticulospinal tract ^4^. Witham *et al*. ^5^ used single intracortical stimuli to activate ‘new’ versus ‘old’ M1 in anaesthetised macaques. Then the authors made intracellular recordings from forelimb and hand motoneurons and found some fast, short-latency monosynaptic responses from ‘new’ M1; no such responses were found from ‘old’ M1. Long-latency monosynaptic excitation, presumably mediated by more slowly conducting CM neurons, was far more common. We know that CM cells have a wide range of soma sizes ^2^ and are certainly not derived solely from the largest (Betz) cells. Long-latency monosynaptic effects were evoked from both regions of M1, as were other more complex, oligosynaptic effects.

The extent of the CM projection in humans, which has been investigated by using non-invasive cortical stimulation ^6, 7^, may well be more widespread than in non-human primates. For example, there is new evidence for a CM projection from ventral premotor cortex in humans ^8^, which in macaques gives rise to corticospinal but not CM projections.

## Quantification of the cortico-motoneuronal output

In 2013, the extent of the CM projection from the hand area of primary motor cortex was quantified by Morecraft *et al*. ^9^. They first identified the hand/arm regions of macaque M1 by intracortical microstimulation (ICMS) and then made injections of anterograde tracers into the cortex close to the central sulcus, and much of the injection site involved the anterior bank of the sulcus. After a recovery period, they sacrificed the animals and carried out a detailed stereological analysis of labelled boutons in the contralateral and ipsilateral spinal cord, analysing tissue taken from C5 to T1 spinal segments. The main conclusions were that almost all labelled boutons (98%) were found in the contralateral cord. This agrees with the finding that stimulation of the pyramidal tract on one side rarely evokes any postsynaptic effects on upper limb motoneurons ^10^. Fibres from hand/arm M1 that terminated contralaterally gave only sparse labelling in the dorsal horn laminae. The largest proportion of contralateral labelled boutons was in lamina VII (59%), confirming a heavy projection to the intermediate zone. However, the second highest proportion of boutons was found amongst the motor nuclei of lamina IX (18%), suggesting that the CM projection is a very significant component of the total corticospinal projection, influencing motoneurons innervating flexors acting on the shoulder and elbow rostrally (C5–C7), along with flexors, extensors, abductors and adductors acting on the digits, hand and wrist caudally (C8–T1). It should be stressed that these motoneurons have widespread dendritic trees that extend well into the intermediate zone, including lamina VII ^11^, which could mean that many of the boutons in that lamina labelled after M1 injections are still CM boutons, terminating on proximal dendrites of target motoneurons.

A somewhat different picture emerges for the corticospinal projection from the leg area of M1, where around 10% of the fibres terminate ipsilaterally, especially among motoneurons of more proximal leg muscles, confirming a number of physiological studies showing bilateral effects on the lower limb ^12^. ****

Rather than defining a neuron by a single target with which its axon establishes a synaptic connection (for example, projection to spinal motoneurons), one should recognize that most central nervous system neurons have axons which arborise to contact multiple targets, the sum of which represents the neuron’s ‘connectome’. To date, most of this work has involved rodents, in which it is possible to use genetic manipulations to recognize all parts of a neuron’s connectome. There is some evidence that the corticospinal connectome is more restricted in primates than in rodents; for example, the cortico-striatal projection appears to be separate from the corticospinal projection in macaques but is shared in rodents ^13^. Again, in primates, a significant part of the corticopontine projection is quite separate from the corticospinal projection, which is much less marked in rodents ^14^.

## Functional relationship between cortico-motoneuronal cell activity and target muscles

Although CM cells have been identified in many different studies, using the method of spike-triggered averaging ^15, 16^, the contribution made by CM cell discharge to the activity of its target muscles, and the postures and movements in which those muscles are recruited, is far from simple ^17^. Whereas approaches to the analysis of populations of motor cortical activity have become more and more sophisticated, how these dynamic representations lead to precise patterning of muscle output is not clear.

Some CM cells do appear to behave like ‘upper motoneurons’ in that their activity closely parallels that of their target muscles ^18, 19^. However, in many cases, CM cell activity can be clearly dissociated from that of their target muscles ^17– 19^. So, for example, Muir and Lemon ^20^ showed that macaque CM cells facilitating intrinsic hand muscles showed strong task-specific effects and that both CM cell and target muscle were active during one task (precision grip) but not during another (power grip). In the latter task, the CM cell was deactivated while the muscle was still active and therefore clearly driven by inputs other than the CM cell being tested.

CM cells rarely facilitate single muscles, and most have a complex ‘muscle field’ which often includes a number of functional synergists ^15, 21, 22^. In a recent study, Griffin *et al*. ^22^ recorded CM cells during performance of a forelimb task in which it is possible to dissociate cortical activity which is ‘muscle-like’ (that is, resembles the timing and pattern of muscle activity) from that which is ‘extrinsic-like’ (that is, resembles the direction of movement produced, independent of arm posture). They found that nearly all of 40 CM cells investigated were ‘muscle-like’. However, it was relatively uncommon for a CM cell to exhibit a pattern of activity similar to that of its target muscle when this was employed as a simple agonist. These authors concluded that the broad distribution of the cell-target muscle vectors, found during performance of the task in three different forearm postures, were such that agonist, synergist, fixator and antagonist functions of target muscles were each well represented by the activity of different CM cells. Indeed, CM outputs organised along these lines show how this system operates to provide a great deal of functional flexibility in the manner of muscle recruitment, a flexibility that cannot be afforded by the relatively fixed synergies represented in spinal motor mechanisms.

Joint fixation can be achieved by co-contraction of antagonistic muscle groups and is crucial for stabilisation of the long, bony articulatory chain from proximal arm to distal phalanx ^23^. The role of the CM system as a muscle fixator has probably been underplayed compared with its role during dynamic, individuated movements. However, many CM cells show sustained activity during the ‘hold’ period of motor tasks ^18, 24^ and so their inputs may be important for maintaining the muscle set for that task.

Studies looking at the natural activity of CM cells are still needed to understand how they contribute to the executed movement. Old-fashioned electrical or modern optogenetic stimulation are extremely useful adjuncts to this approach but cannot simulate the natural activity of CM cells. For example, recent work suggests that long trains of ICMS, rather than simulating motor cortex output, give misleading accounts of motor organisation because they ‘hijack’ intracortical circuits and override normal patterns of CM output ^25^.

Even single intracortical shocks can evoke high-frequency, repetitive firing from corticospinal neurons ^26^. Therefore, the results are possibly more difficult to interpret than the effects seen in spike-triggered averages based on the natural, movement-related discharge of CM cells. Nevertheless, careful use of single-pulse ICMS by Cheney *et al*. has been important in determining the complex maps of muscle outputs in primary ^27, 28^ and secondary ^29, 30^ motor areas. These studies have helped to demonstrate the direct, short-latency influence of M1 over forelimb muscles, compared with slower effects evoked from secondary motor areas.

## Fast and slow conduction in the corticospinal tract

The primate corticospinal tract (CST) is remarkable in displaying a 100-fold range of axon diameters ^31^. Most mammals have a very large number of small corticospinal fibres, ranging from around 3 µm to as small as 0.5 µm ^4, 32^. Larger primates (including the macaque, spider monkey, gibbon and human) also possess large-diameter fibres, up to around 12 µm in macaques and up to 22 µm in humans. Body size alone is clearly not the key factor since much larger mammals such as cows and whales have relatively small corticospinal fibres. A number of different explanations have been put forward for large, fast axons in primates, including the branching pattern of the ‘connectome’, the need to maintain high firing rates, and the importance of reducing conduction delays during transitions from movement to posture in skilled grasp ^33, 34^.

These fast fibres are very much in the minority, comprising a small percentage of the total, but with an importance out of all proportion to their numbers. (It has been estimated that, in the macaque tract with around 600,000 fibres, about 18,000 fibres have diameters greater than 3 µm ^31^.) The larger fibres are particularly vulnerable to disease and trauma (for example, during spinal cord injury) ^35, 36^. M1 gives rise to some of the fastest fibres, and there is a bias towards faster fibres from ‘new’ versus ‘old’ M1 ^5, 37^. Long-latency monosynaptic effects generated by M1 stimulation ^5^ had latencies up to around three or four times longer than that of the fastest CM effects, so if these fastest effects were conducted by axons with conduction velocities around 70 m/s, the slowest effects would derive from axons conducting at around 17 m/s, still well above the velocity (~7 m/s) for the median axon diameter of M1 corticospinal neurons of around 1.2 µm ^37^.

There is a problem in understanding the function of the slowest fibres; they are largely missing from neurophysiological studies in primates using antidromic activation of CST neurons from the pyramidal tract ^5, 31, 38^, and there are a number of possible explanations for this ^39^. Until a better means of electrophysiologically identifying the neurons giving rise to the slowest CST fibres can be found, the function of these slow neurons will remain a mystery.

## The cortico-motoneuronal system in the wider context of cortical control of movement

It has been emphasised that the CM system does not work alone but rather in concert with other descending and spinal segmental systems ^40, 41^. Nevertheless, there is accumulating evidence that the CM system adds the characteristic capacity for skilled hand movements, including the ‘individuation’ of finger movements ^40^. Early, classic studies showed that complete pyramidotomy permanently abolished relatively independent finger movements (RIFMs) ^42– 44^. However, if the pyramidotomy was incomplete, there was often substantial recovery ^43^, suggesting that sparing a relatively small proportion of the total corticospinal and CM outflow can be the basis of a return of hand function.

Interruption of the lateral (crossed) CST at different spinal cervical levels also abolishes RIFM, but again there is some degree of recovery ^45, 46^. Since corticospinal fibres make many different types of connections within the spinal cord, it is a continuing challenge to determine which of them makes critical contributions to RIFM. In addition to their CM connections, CST fibres give rise to inputs to C3–C4 propriospinal neurons which send descending projections to forelimb motoneurons located in the lower cervical segments. CST fibres exert further indirect excitatory and inhibitory effects via interneurons in these same lower segments, and these interneurons appear to receive the bulk of corticospinal input from M1 ^9^. A lesion of the lateral CST at the mid-cervical level interrupts all of the CM and other corticospinal inputs to lower cervical segments but leaves the descending axons of C3–C4 propriospinal neurons intact ^45^. It is noteworthy that such a lesion results in a devastating initial loss of the precision grip needed by the monkey to retrieve small rewards ^45, 47, 48^. This is consistent with the notion that the CM system plays a major role in the execution of precision grip in the intact animal.

After this initial deficit, recovery occurs over a period of a few weeks. This recovery has been shown to involve the C3–C4 system ^45, 47, 48^. It has also been demonstrated that, although precision grip performance returns to control levels, there are subtle differences in the pattern of electromyographic activity, suggesting that changes in control have taken place to compensate for the loss of CM inputs ^45^.

Damage to the human motor cortex and CST is generally far more devastating than in animal models and this may be because the CM system is best developed in humans ^40^. Interestingly, classic studies of the effects of mid-cervical cordotomy for pain relief in humans showed that lesions of the lateral funiculus which avoided the CST but which would have interrupted more ventrally located propriospinal fibres did not affect the function of upper limb movement ^49^.

An important study by Zaaimi *et al*. ^10^ showed that recovery after pyramidal lesions probably involves marked upregulation in reticulospinal control of hand motor nuclei. In the intact macaque, these inputs overlap with those from the corticospinal system ^50^, although they are significantly weaker. However, 6 months after a unilateral pyramidal lesion, there were considerably higher levels of reticulospinal excitation of forelimb flexor and intrinsic hand motoneurons ^10^, which may well contribute to the recovery of crude grasp after a pyramidal lesion or cortical stroke ^51^. These results are of great importance in trying to disentangle the positive and negative motor signs after a stroke in humans and in designing new therapies to enhance the reticulospinal contribution to recovery of hand function ^52^.

## The cortico-motoneuronal system, dexterity, and new findings from the mouse

CM connections are particularly well developed in dexterous, tool-using primates. In a recent study, Quallo *et al*. ^24^ demonstrated that macaque corticospinal neurons, including some CM cells, were just as active during tool use, involving use of a rake to retrieve food rewards, as they were during a precision grip. It might well be expected that the same neurons that are involved in control of skilled hand and finger movements are also recruited during tool use; this study was the first to demonstrate that to be the case.

Rodents do not possess CM connections, and their manipulative skills have developed along quite different lines, well suited to their ecological niche. Because of the methodological advantages of using the rat (and particularly the mouse) for the genetic dissection of neural control systems, a number of studies have sought to model grasp in rats or mice by training them to retrieve small pellets with the forepaw. These studies generally show that, even after training, rodents generally achieve quite low levels of success on such tasks, often around 50 to 60% (for example, 53, 54). It is important to stress the fundamental difference between precision grip in the Old World primate, such as the macaque, and the grasp executed by rodents. It is noteworthy that macaques can be trained to perform precision grip at success rates close to 100% ^55^.

Of course, it would be very interesting if ‘dexterity’ in rodents could be improved by supplementing their forepaw control system with CM connections. A fascinating piece of recent research has achieved just this ^54^. This study took advantage of the fact that CM connections, though absent in adult rodents, are transiently present in the neonatal animal, before being withdrawn in the early postnatal period ^54, 56^. Gu *et al*. ^54^ sought to interfere genetically with the plexin-based signalling system involved in withdrawal of CM projections. They developed a PlexA1 mutant in which CM connections established at birth are maintained into adulthood. Other descending pathways were not affected. Using ICMS, the authors demonstrated that these mutants had a fast motor pathway from motor cortex to forelimb muscles, which was lacking in the wild-type mouse. They also showed that the mutant mice could be trained to perform a pellet-grasping task at higher levels of success than wild-type animals. Because other descending pathways were not changed in the mutants, this lends further support to a main role for the corticospinal system in enhancing skilled grasp.

A further fascinating part of this discovery was to demonstrate the existence of a CIS-regulatory system in layer V of motor cortex that inhibits the Plexin signalling and thereby allows CM connections, all of which are derived from layer V corticospinal neurons, to be maintained into adulthood. The authors showed that this inhibitory system is strongly expressed in motor cortex of dexterous primates such as human, chimpanzee, orangutan and baboon but is not present in other less dexterous primates, such as marmoset and bushbaby, or in rats or mice ^54^.

## The cortico-motoneuronal system and motor neuron disease

Although the involvement of the CST in motor neuron disease (amyotrophic lateral sclerosis, or ALS) was known from the earliest descriptions of the disease, accumulating evidence now suggests an especially important role for the CM component of the corticospinal system in some forms of ALS ^57, 58^. For example, a distinctive feature in the early stages of ALS in some patients is the ‘split-hand’ syndrome, in which there is much greater weakness and wasting of the muscles acting on the radial side of the hand (moving the thumb and index finger) than on the ulnar side (hypothenar muscles, acting on the little finger).

The main cause of this striking contrast does not appear to be ALS-related pathology in the peripheral neuromuscular system but rather a TDP43 pathology acting on the CM projection. It is well known that there is a disproportionately large representation of thumb movements in M1, and electrophysiological studies in both non-human primates ^1, 59^ and humans ^6, 7^ have shown that the largest monosynaptic excitation following corticospinal activation is found in intrinsic thumb and index finger muscles, and there are weaker effects on the hypothenar musculature. In early stages of the disease, the impact on CM projections results in specific, task-related deficits, including use of the thenar hand for a multitude of manipulative skills, including writing, typing and doing up buttons.

More widely, ALS pathology involving CM projections might affect skilled aspects of locomotion ^60^ and speech ^58^. It is noticeable that CM projections to the nucleus ambiguus, which innervates the laryngeal musculature, are present in humans but not in monkeys ^61^.

Braak *et al*. have gathered evidence that the early stages of ALS involve TDP43 pathology in cortical layer V pyramidal neurons with long axons projecting to subcortical and spinal targets ^62^. It has been suggested that the TDP43 pathogen is transmitted via these long axons to all target motoneurons of the CM system, which in turn results in the ‘lower motoneuron’ signs characteristic of ALS. Interestingly, recent evidence suggests that primate pyramidal neurons have membrane properties quite different from those in the rodent, including high expression levels of the fast potassium channel, Kv3.1b, which is not found in rat pyramids ^63^. This difference, and probably others, might contribute to the vulnerability of the TDP43 pathology in primates, explaining why the cortical stages of ALS have been more difficult to demonstrate in the rodent ^62^. Understanding the species-specific properties of the primate CM system might provide a valuable model for understanding and treating ALS.

## Conclusions and prospects

The CM system is unique to primates and particularly well developed in humans. It provides a fast, direct pathway to motoneurons, particularly those supplying muscles that subserve some of the most characteristic human hand movements, including those for tool use, music making, gesture and communication. CM control signals effectively bypass the more rigid networks provided by spinal segmental connectivity and support a much richer repertoire of grasping movements, based on the combination and recombination of many different CM outputs to hand and digit muscles. Although our knowledge of the organisation of this system is now much improved, we still need to know more about its functional contributions to skilful movement. In animal studies, a better definition of the molecular and genetic identity of the CM system might provide further clues, including the possibility of a selective CM cell blocker which might give further insights into not only CM function but the recovery process involving other systems. Selective tools that could be used to repair or promote the CM system could be used in therapeutic studies.
